# Perception of burden by caregivers of patients with schizophrenia

**DOI:** 10.4103/0019-5545.55938

**Published:** 2005

**Authors:** Sunil Srivastava

**Affiliations:** * Senior Psychiatrist, Institute of Mental Health and Hospital

**Keywords:** Burden Assessment Schedule, Pearson correlation, biserial correlation coefficient

## Abstract

**Background:**

There is a dearth of studies assessing the burden of caregivers of patients with schizophrenia and illness-related variables such as age, sex, duration of illness, domicile, martial status, education, employment and previous hospitalization.

**Aim:**

The study was conducted to measure the perception of burden by caregivers of patients with schizophrenia and its correlation with nine factors on the Burden Assessment Schedule (BAS) related to spouse, physical and mental health, external support, caregiver's routines, support to patient, responsibility-taking, other relatives, patient's behaviour and caregiver's strategy.

**Methods:**

Using BAS, we assessed the burden in a sample of caregivers of 34 patients with schizophrenia taken from the OPD of the Institute of Mental Health and Hospital, Agra.

**Results:**

A low positive correlation was found between urban domicile and support of the patient; of domicile Agra and effect on other relations; and domicile Agra and effect on the caregiver's routine. There was a low positive correlation between age less than 30 years and the physical and mental health of the caregiver, and with taking responsibility. The *t* test for population correlation was significant up to 5% probability level (p<0.05) for correlation between urban domicile and support of the patient; between domicile Agra and effect on other relations; between domicile Agra and the effect on the caregiver's routine; between age less than 30 years and the physical and mental health of the caregiver; and between age less than 30 years and taking responsibility.

**Conclusion:**

Further studies in this field are required including one with a non-linear correlation analytic design.

## INTRODUCTION

Severe mental disorders such as schizophrenia have far-reaching consequences.^1^,^2^ Evidence suggests that family members experience significant stress in coping with a person with schizophrenia.^3^ The patient's relatives experience feelings of loss and grief.^4^ They are confronted with uncertainty and emotions of shame, guilt and anger. Like the patient, they feel stigmatized and socially isolated.^5^ Their lives may be disrupted by providing more care than would be normal for someone of the patient's age. In cases where the reciprocity between family members is out of balance, normal care changes to caregiving. Addition of the caregiving role to the already existing family role may become stressful, both psychologically and economically.^6^,^7^

Horowitz and Reinhard^8^ differentiate between caregiver duties (which encompass the involvement and responsibility of caregivers) and caregiver burden (which they define as the consequence that caregiving activities have for families). Treudley^9^ referred to burden as the consequence for those in close contact with a severely ill psychiatric patient. The chronic illness of a family member is considered an objective stressor that, because of the caregiving tasks, results in a strain for the caregiver or the relative. The consequences for a patient's relatives, formerly referred to as family or caregiving burden, have been studied for almost four decades.^10^,^11^ Initially, the studies were purely descriptive. In the early 1970s, instruments were developed^12^ and subsequently used in epidemiological studies and randomized clinical trials.^13^–^16^ Several researchers have identified factors that adversely influence the experiences of carers—psychological and emotional distress; physical illness; disruption of the family, and social and sexual relationships; curtailment of social activities; and financial hardship. The chronic burden of caregiving to a patient with schizophrenia is likely to generate negative emotions and their expression. Expressed emotion (EE) comprises critical or emotionally over-involved attitudes and behaviours.^17^ A meta-analysis showed a 48% median relapse rate in a high EE environment versus 21% in a low EE environment.^18^ Higher subjective levels of burden and personal stress have been reported by high EE relatives compared with low EE relatives.^19^ For many carers, frustration, anger, loneliness and despair are common.^20^–^22^ Levene *et al.*^3^ reported that the Perceived Family Burden Scale, an instrument for measuring patient behaviour and family burden, demonstrated predictive power for early symptomatic relapse in schizophrenia.

It is, therefore, observed that the perceived burden has a serious impact on the caregiver's physical and emotional health; social relationships and perception; and expression of negative emotions such as frustration, despair, loneliness and anger, which have an influence on the course of the schizophrenic illness of the patient under care. There is a dearth of studies assessing the burden of caregivers of patients with schizophrenia and illness-related variables such as age, sex, duration of illness, domicile, marital status, education, employment and previous hospitalization. Therefore, this study was undertaken to find out, using the Burden Assessment Schedule (BAS) by Thara *et al.,*^23^ whether there is any correlation between the perceived burden of caregivers of patients with schizophrenia and age, sex, duration of illness, marital status, previous hospitalization, education, domicile and employment status of the patients.

## METHODS

The study sample was drawn from patients diagnosed to be suffering from schizophrenia by a psychiatrist as per the ICD-10 criteria and attending the OPD of the Institute of Mental Health and Hospital, Agra, in the month of November 2003 on Mondays, Wednesdays and Fridays.^24^ Patients having concurrent physical illness or those brought by the police or transferred from protection homes or orphanages, and patients of non-consenting caregivers were excluded from the study. Data including details of the present history, family history and pre-morbid personality of the patients were recorded on a pre-designed proforma. Thereafter, the identification and personal history details of the caregiver of the patient were recorded. The perception of burden of the caregiver was measured using BAS.^23^ The schedule contains 40 questions rated on a three-point scale marked from 1 to 3. The responses are ‘not at all’, ‘to some extent’ or ‘very much’. The scale has nine factorial configurations—spouse-related, physical and mental health, external support, caregiver's routines, support of the patient, taking responsibility, other relations, patient's behaviour and caregiver's strategy. The items are contextual and reflect the opinion of the caregiver. The point biserial correlation coefficient was used to find out the correlation between one variable being continuous (the Burden assessment Schedule score in this study) and the other being a nominal variable with two classification levels, e.g. male–female, employed–unemployed, etc. The biserial correlation coefficient has been calculated for one variable being quantitative and the other being dichotomous, but with underlying continuity. For point biserial analysis, 1 was assigned to patients being employed, unmarried, male, belonging to Agra, having a past history of admission to a mental hospital and having an urban domicile. For biserial correlation, 1 was assigned for age <30 years and duration of illness ≤6 months. Alternate categories have been placed in the zero score category; *t* test was used to find out the statistical significance of the correlation obtained.

Data on the sociodemographic profile of the study group were recorded, including the duration of illness and past history of admission to a mental hospital (Table 1). The majority of subjects were in the 20–49 years' age group (85%). About three-fourths of the subjects were married (*n*=26; 76%). To study the influence of distance and related transportation problem on the felt burden, we noted whether the patients were from or outside Agra. Only 5 subjects were from Agra (15%). Twenty-four patients (71%) were literate whereas only 10 were illiterate (29%). There was a history of past admission in a psychiatric hospital in 8 patients (24%). The nine categories of BAS are shown in Table 2.

**Table 1:** Structure of the study sample of patients with schizophrenia (*n*=34)

Variable	*n* (%)
Age (years)	
<20	3 (9)
20–29	14 (41)
30–39	9 (26)
40–49	6 (18)
50–59	2 (6)
>59	0
Sex	
Male	23 (68)
Female	11 (32)
Marital status	
Single	8 (24)
Married	26 (76)
Widow/widower	0
Separated	0
Employment status	
Employed	8 (24)
Unemployed	26 (76)
Domicile	
Of Agra	5 (15)
Not of Agra	29 (85)
Urban	10 (29)
Rural	24 (71)
Duration of illness	
≤ 6 months	20 (59)
>6 months	14 (41)
Educational status	
Literate	24 (71)
Illiterate	10 (29)
Previous history of admission to a mental hospital	
Past history of admission to a mental hospital	8 (24)
No previous history of admission to a mental hospital	26 (76)

**Table 2:** Categories on the Burden Assessment Schedule (BAS)

Factor number	Category
Factor 1	Spouse-related
Factor 2	Physical and mental health
Factor 3	External support
Factor 4	Caregiver's routines
Factor 5	Support of the patient
Factor 6	Taking responsibility
Factor 7	Other relations
Factor 8	Patient's behaviour
Factor 9	Caregiver's strategy

## RESULTS

### 1. Age

A low positive correlation was found between age <30 years and factor 2, and between the same age group and factor 6 (Table 3).

**Table 3:** Correlation of age with each factor of the Burden Assessment Schedule

	Correlation coefficient (r)	Significance level
Age <30 years vs factor 1	0.11	p>0.05
Age <30 years vs factor 2	0.41	p<0.01
Age <30 years vs factor 3	0.17	p>0.05
Age <30 years vs factor 4	0.09	p>0.05
Age <30 years vs factor 5	0.27	p>0.05
Age <30 years vs factor 6	0.35	P<0.05
Age <30 years vs factor 7	0.11	p>0.05
Age <30 years vs factor 8	0.15	p>0.05
Age <30 years vs factor 9	0.16	p>0.05

### 2. Marital status

Marital status was found to have little, if any, correlation with any of the factors of BAS. Similarly, the sex of the patient did not have a significant correlation with any of the factors of BAS.

### 3. History of past admission

History of past admission was not found to have any significant correlation with any of the factors of BAS.

### 4. Literacy

The educational level of the patient did not have any significant correlation with any of the factors of BAS.

### 5. Domicile

Patients having an urban domicile were found to have a low positive correlation with factor 5; the remaining factors showed no significant correlation with urban domicile (Table 4). Factor 4 showed a low positive correlation with Agra domicile (Table 5).

**Table 4:** Correlation between urban domicile and factors on the Burden Assessment Schedule

	Correlation coefficient (r)	Significance level
Urban domicile vs factor 1	0.23	p>0.05
Urban domicile vs factor 2	0.07	p>0.05
Urban domicile vs factor 3	0.06	p>0.05
Urban domicile vs factor 4	0.01	p>0.05
Urban domicile vs factor 5	0.32	p<0.05
Urban domicile vs factor 6	0.12	p>0.05
Urban domicile vs factor 7	0	p>0.05
Urban domicile vs factor 8	0.26	p>0.05
Urban domicile vs factor 9	0.03	p>0.05

**Table 5:** Correlation between domicile (Agra/non-Agra) and factors on the Burden Assessment Schedule

	Correlation coefficient (r)	Significance level
Domicile Agra vs factor 1	0.21	p>0.05
Domicile Agra vs factor 2	0.14	p>0.05
Domicile Agra vs factor 3	0.18	p>0.05
Domicile Agra vs factor 4	0.45	p<0.01
Domicile Agra vs factor 5	0.18	p>0.05
Domicile Agra vs factor 6	0.04	p>0.05
Domicile Agra vs factor 7	0.21	p<0.05
Domicile Agra vs factor 8	0.02	p<0.05
Domicile Agra vs factor 9	0.04	p>0.05

### 6. Duration of illness

A duration of illness of ≤6 months was found to have no correlation with any of the factors of BAS. Similarly, the employment status of patients did not have any significant correlation with any of the factors of BAS.

## DISCUSSION

A majority of the patients were males (68%), married (76%), unemployed (76%), had a rural domicile (71%), were from outside Agra (85%), literate (71%), and without any past history of admission to a mental hospital (76%). The majority of patients (41%) belonged to the 20–29 years' age group.

A low positive correlation between age <30 years and factor 2, i.e. physical and mental health of the caregiver, and with factor 6, i.e. taking responsibility, may be indicative of a heavy psychological and physical strain on the caregiver and his/her responsibility-taking behaviour of patients with schizophrenia in their formative or early productive period.

Schene *et al.*^25^ did not find a significant correlation between the age of patient and the caregiver's domains of tension or worry, but found an item-total correlation for factors about financial worries regarding the patient's management; concern about future finance is one of the items in the responsibility-taking factor.

The low positive correlation which comprises current financial resources to care for the patient, reduced time spent with the patient, and caregiver forced to work to support the patient may be reflective of the perception of financial strain of the urban caregiver.

The domiciliary treatment of patients with schizophrenia, particularly of those having illness for ≤6 months (59%), entails a heavier burden on the caregiver. It appears from the observations that a higher BAS score reflects this trend. The correlation between the duration of illness and the BAS score may be associated with illness-related factors as shorter-duration schizophrenia may present more taxing management problems compared with chronic schizophrenia. Several studies^26^–^28^ have reported positive symptoms, more common in short-duration schizophrenia, to cause greater burden on the caregiver. Apathy has been found to be not related to caregiver's tension, supervision and worrying.^25^ As 76% of the patients did not have a past history of admission to a mental hospital, the result/finding may also reflect the factor of inexperience.

The very low positive correlation of literacy with BAS scores (r=0.23, coefficient of determination=0.0529) may be due to the influence of the sample structure as 71% of the patients were literate. Contrary to the expected, being employed had little correlation with the burden scores, whereas a loss of earning due to a major psychiatric illness is likely to be perceived as an added burden. It needs to be interpreted with caution as the coefficient of determination is low and 76% of the patients were unemployed. With 68% of the patients being men and the correlation coefficient between being male and BAS score being 0.19, the association is very weak and may be a sample bias,^29^ but the trend is in keeping with the finding of Thara and Joseph^30^ that men tend to be more disabled in occupational functioning and the role of women is ill defined.

Occupational impairment appears to be a factor for a gender-related differential trend of BAS score. Our study shows little, if any, correlation between being of rural domicile and the BAS score. Mubarak Ali and Bhatti^31^ have found that the burden perceived by relatives of a patient with chronic schizophrenia was the same in urban and rural families. The present study failed to indicate a significant correlation between past history of admission to a mental hospital. Bailer *et al.*^32^ reported an association between high-rejecting attitude of family members and the number of hospitalizations in patients with schizo-phrenia. As our sample consisted mainly of patients without a past history of hospitalization (76%), the lack of an association may be reflective of the sample structure.

In a discussion on the findings of the study with the psychiatrists at the Institute of Mental Health and Hospital, it was generally felt that caregivers of very young or old patients with schizophrenia felt a lesser burden of caregiving as compared with caregivers of middle-aged patients. The positive results of this study are only weak indicators as the level of correlation is a very low positive and the correlation is unlikely to hold true for the entire population (p>0.05). The results, therefore, indicate little or no linear correlation between the identified variables and the felt burden. This study therefore highlights the need for further studies in this area, especially ones with non-linear correlation analytical designs.
